# Somatic genomic mosaicism in the brain during aging: Scratching the surface

**DOI:** 10.1002/ctm2.1138

**Published:** 2022-12-10

**Authors:** Taejeong Bae, Yifan Wang, Flora M Vaccarino, Alexej Abyzov

**Affiliations:** ^1^ Department of Quantitative Health Sciences Center for Individualized Medicine, Mayo Clinic Rochester Minnesota USA; ^2^ Child Study Center Yale University New Haven Connecticut USA; ^3^ Department of Neuroscience Yale University New Haven Connecticut USA

1

Mutagenesis occurs in human cells starting from the fertilized egg and continuing throughout life, resulting in somatic mutations.^1^ Most somatic mutations are functionally benign and have neither harmful nor beneficial effects on health. In rare cases, they change cell functions and may lead to diseases. Cancer is the most common example of a genetic disorder caused by somatic mutations. In recent years, interest has been increasing in the roles of somatic mutations in other diseases, including neuropsychiatric diseases such as autism and schizophrenia.^2^, ^3^ But their causal relationships with somatic mutations remain unclear. To investigate the role of somatic mutations in the development of neuropsychiatric disorders, the Brain Somatic Mosaicism Network (BSMN) was formed as a consortium effort through the support of the National Institute of Mental Health.^4^

In our recent study, in which the Mayo Clinic, Yale University, and the Lieber Institute for Brain Development worked together within the frame of the BSMN, we analyzed 131 post‐mortem human brains from 44 healthy individuals, 19 with Tourette syndrome, nine with schizophrenia and 59 with autism spectrum disorder.^5^ The study reported several interesting findings by whole‐genome sequencing of the brains to a depth of over 200X. First, most brains had 20–60 detectable single‐nucleotide mutations that likely arose in early development. There were no differences in the somatic mutation burden between diseased and normal brains. Unexpectedly, seven brains, about 6% of the total, carried an abnormally large number—at least 100 but as many as 2000—of somatic mutations. This phenomenon was termed hypermutability.

Upon closer examination of the hypermutable brain with the highest number of somatic mutations, LIBD82, it was found that about 15% of hippocampal cells in this brain harbored a duplication of chromosome 7 and a deletion of chromosome 10. Reanalysis of the data for this brain with a focus on the telomerase reverse transcriptase (TERT) promoter revealed a canonical mutation (G > A on chr5:1295250) in a similar fraction of cells in the hippocampus. Concurrent gain of chromosome 7, loss of chromosome 10 and mutation in the TERT promoter are molecular characteristics commonly observed in glioblastoma, a fast‐growing and aggressive brain tumor.^6^, ^7^ These findings indicate that the LIBD82 brain likely carried an undiagnosed early‐stage brain cancer, although it came from a schizophrenia patient with no prior clinical cancer history. Therefore, the hypermutable phenotype of this brain probably reflects an in vivo clonal expansion during tumor development. Through clonal expansion, all somatic mutations in a specific cell lineage increase their frequencies to a detectable level in bulk brain tissue. Interestingly, 10 damaging mutations in cancer‐driving genes were found in four of the other six hypermutable brains, therefore implying clonal expansion in those brains as well. Consistently, hypermutability is typically localized to one brain region, although that estimate could be biased as no more than two regions per brain were analyzed. The hypothesis of clonal expansion was confirmed in hypermutable brain NC7, in which somatic mutations had higher allele frequency in the interneuron fraction from the striatum. Analysis of the genomes of single cells from that fraction proved the existence of a clonal cell population encompassing ∼50% of all cells.

The question of which cell type expanded remains open (see Figure 1). It could be interneurons, as the experiment in NC7 suggests. In such a case, lineage expansion occurs in neural precursors before they fully differentiate into post‐mitotic interneurons that migrate and populate the brain during prenatal development.^8^ However, since the cell fractionation experiment may not isolate pure interneurons, clonally expanded cells could theoretically be some other cell type. Unlike post‐mitotic neurons, glial cells continue to divide in adult brains. Given previous studies reporting increased glial cell fraction with age,^9^, ^10^, ^11^ the hypothesis of clonal gliogenesis is consistent with the observation that hypermutable brains are older. Clonal hematopoiesis with infiltration of blood into the brain could be yet another possibility. This hypothesis emerges from the observation that mutated cancer‐driving genes in hypermutable brains are frequently associated with clonal hematopoiesis. In aging adults, clonal hematopoiesis increases,^12^ and the blood–brain barrier also becomes leakier.^13^ So, expanded cell lineages in the blood could be detected in the brain. Further studies are required to determine which hypothesis is correct, but it is possible that all could be correct, and there could be different causes of hypermutability in different individuals.

**FIGURE 1:**
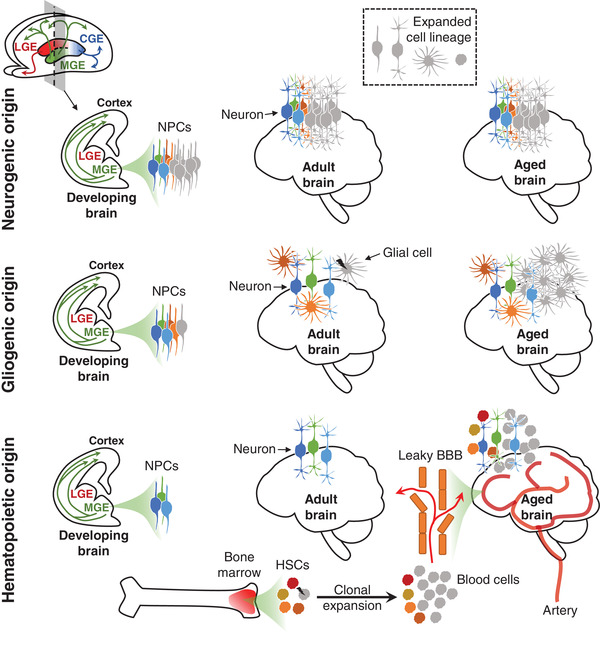
Three hypotheses for the origin of clonally expanded cell population in hypermutable brains. BBB, blood‐brain barrier; CGE, caudal ganglionic eminences; HSC, hematopoietic stem cell; LGE, lateral ganglionic eminences; MGE, medial ganglionic eminences; NPC, neural progenitor cell

Hypermutability increased with age, reaching ∼16% among old brains (>60 years of age), while it was only ∼2% among younger brains (<40 years of age). As hypermutability was observed in the brains of healthy individuals and in those with neuropsychiatric diseases, no link of hypermutability to neuropsychiatric disease states has been found, nominating age as the primary causal factor. Age is known to be the major factor associated with cancer occurrence. As such, the observed hypermutability carries two major hallmarks of cancers: clonal expansion and age association. This suggests that hypermutability generally represents pre‐cancer or undiagnosed cancer cases, implying a theoretical possibility of cancer monitoring and early detection based on genomics. Association with aging also implies that hypermutability could be relevant in other aging‐associated diseases such as Parkinson's and Alzheimer's. If proven, they theoretically could be diagnosed early before symptoms develop, using hypermutability as a genomic biomarker. However, it is currently unclear how this could be achieved, given that brain tissue is hardly accessible.

Among other findings, no significant differences between diseased and normal brains were observed in the proportions of somatic mutations in different categories of genomic annotation (e.g., coding, noncoding). However, in the brains of individuals with autism—as compared to neurotypical ones—somatic single‐nucleotide mutations generated more motifs for transcription factor binding within active enhancers of the developing brain. The most noticeably affected motifs were binding sites for myeloid ecotropic viral integration site (MEIS) homeodomain transcription factors, which are known to be involved in embryonic development.^14^ In addition, somatic structural mutations and copy number alterations were unexpectedly frequent and found in about 7% of the brains. Most of them (in about 5% of the brains) were somatic duplications, which are unlikely to have functional changes and likely reflect background mutagenesis.^5^

The study made possible by the BSMN revealed the presence of hypermutable brains and insights into the possible role of somatic mutations in autism spectrum disorder. However, despite a significant effort, the studied cohort is still relatively small and at least 100 times smaller than in modern GWAS studies, limiting the discovery of the possible role of somatic mutations in neuropsychiatric diseases. We believe that further studies with larger cohorts will yield more insights.

## References

[1] Manders F , van Boxtel R , Middelkamp S . The dynamics of somatic mutagenesis during life in humans. Front Aging. 2021;2:802407. doi:10.3389/fragi.2021.802407 35822044 PMC9261377

[2] Vijg J , Dong X . Pathogenic mechanisms of somatic mutation and genome mosaicism in aging. Cell. 2020;182:12‐23. doi:10.1016/j.cell.2020.06.024 32649873 PMC7354350

[3] Bizzotto S , Walsh CA . Genetic mosaicism in the human brain: from lineage tracing to neuropsychiatric disorders. Nat Rev Neurosci. 2022;23:275‐286. doi:10.1038/s41583-022-00572-x 35322263

[4] McConnell MJ , Moran JV , Abyzov A , et al. Intersection of diverse neuronal genomes and neuropsychiatric disease: the Brain Somatic Mosaicism Network. Science. 2017;356:eaal1641. doi:10.1126/science.aal1641 28450582 PMC5558435

[5] Bae T , Fasching L , Wang Y , et al. Analysis of somatic mutations in 131 human brains reveals aging‐associated hypermutability. Science. 2022;377:511‐517. doi:10.1126/science.abm6222 35901164 PMC9420557

[6] Crespo I , Vital AL , Nieto AB , et al. Detailed characterization of alterations of chromosomes 7, 9, and 10 in glioblastomas as assessed by single‐nucleotide polymorphism arrays. J Mol Diagn. 2011;13:634‐647. doi:10.1016/j.jmoldx.2011.06.003 21884817 PMC3194060

[7] Nonoguchi N , Ohta T , Oh J‐E , et al. TERT promoter mutations in primary and secondary glioblastomas. Acta Neuropathol. 2013;126:931‐937. doi:10.1007/s00401-013-1163-0 23955565

[8] Lim L , Mi D , Llorca A , Marín O . Development and functional diversification of cortical interneurons. Neuron. 2018;100:294‐313. doi:10.1016/j.neuron.2018.10.009 30359598 PMC6290988

[9] Conde JR , Streit WJ . Microglia in the aging brain. J Neuropathol Exp Neurol. 2006;65:199‐203. doi:10.1097/01.jnen.0000202887.22082.63 16651881

[10] Diniz DG , Foro CAR , Rego CMD , et al. Environmental impoverishment and aging alter object recognition, spatial learning, and dentate gyrus astrocytes. Eur J Neurosci. 2010;32:509‐519. doi:10.1111/j.1460-9568.2010.07296.x 20704596

[11] Tremblay M‐È , Zettel ML , Ison JR , et al. Effects of aging and sensory loss on glial cells in mouse visual and auditory cortices. Glia. 2012;60:541‐558. doi:10.1002/glia.22287 22223464 PMC3276747

[12] Jaiswal S , Ebert BL . Clonal hematopoiesis in human aging and disease. Science. 2019;366:eaan4673. doi:10.1126/science.aan4673 31672865 PMC8050831

[13] Banks WA , Reed MJ , Logsdon AF , et al. Healthy aging and the blood‐brain barrier. Nat Aging. 2021;1:243‐254. doi:10.1038/s43587-021-00043-5 34368785 PMC8340949

[14] Schulte D , Geerts D . MEIS transcription factors in development and disease. Development. 2019;146:dev174706. doi:10.1242/dev.174706 31416930

